# Intractable Headaches, Ischemic Stroke, and Seizures Are Linked to the Presence of Anti-β2GPI Antibodies in Patients with Systemic Lupus Erythematosus

**DOI:** 10.1371/journal.pone.0119911

**Published:** 2015-03-17

**Authors:** Tomasz Hawro, Andrzej Bogucki, Maria Krupińska-Kun, Marcus Maurer, Anna Woźniacka

**Affiliations:** 1 Department of Dermatology and Allergy, Charité—Universitätsmedizin Berlin, Berlin, Germany; 2 Department of Extrapyramidal Diseases, Medical University of Łódź, Łódź, Poland; 3 Department of Affective and Psychotic Disorders, Medical University of Łódź, Łódź, Poland; 4 Department of Dermatology and Venereology, Medical University of Łódź, Łódź, Poland; Instituto Nacional de Ciencias Medicas y Nutricion Salvador Zubiran, MEXICO

## Abstract

**Background:**

Neuropsychiatric systemic lupus erythematosus (NPSLE) is a common and potentially fatal manifestation of SLE. Antiphospholipid antibodies (aPL) such as lupus anticoagulant (LA), anticardiolipin (aCL) and antibodies to β2glycoprotein I (anti-β2GPI), the most important aPL antigen, are thought to play a role in some forms of NPSLE. As of yet, their specific roles in NPSLE manifestations remain to be elucidated.

**Methodology/Principal Findings:**

57 SLE patients (53 women) were assessed for LA, aCL and anti-β_2_GPI twice, to determine persistent positivity. All patients were examined by neurology and psychiatry specialists. 69 healthy subjects were assessed as controls. NPSLE was diagnosed in 74% of patients. Headaches were the most prevalent manifestation of NPSLE (39%), followed by cerebrovascular disease (CVD) (23%), depressive disorders (19.0%), and seizures (14%). NPSLE and non-NPSLE patients showed comparable SLE activity and corticosteroid use. In 65% of patients neuropsychiatric manifestations preceded SLE diagnosis. aPL profiles of NPSLE patients and non-NPSLE patients were similar. Headaches and ischemic stroke were independently associated with anti-β_2_GPI-IgM (OR=5.6; p<0.05), and seizures were linked to anti-β_2_GPI-IgG (OR=11.3; p=0.01).

**Conclusions:**

In SLE patients, neuropsychiatric manifestations occur frequently and early, often before the disease is diagnosed. Autoantibodies to β2GPI are linked to non-specific headaches, ischemic stroke and seizures, and show a better predictive value than aCL and LA. These findings may help to improve the diagnosis of NPSLE and should prompt further studies to characterize the role of anti-β2GPI in the pathogenesis of this condition.

## Introduction

Systemic lupus erythematosus (SLE) is a chronic, multisystem, autoimmune disease with autoantibody-mediated tissue damage. Clinically, SLE is characterized by heterogeneous symptoms and may involve almost all tissues and organs, including the nervous system. Neuropsychiatric lupus encompasses a wide spectrum of neurologic and psychiatric disorders resulting from the involvement of the central, peripheral and autonomic nervous system due to SLE-related pathology. The attribution of different neurologic and psychiatric disorders to neuropsychiatric SLE (NPSLE) is still a matter of debate. NPSLE is a relatively frequent and potentially fatal presentation of SLE, and the involvement of CNS especially is associated with a more serious course and increased mortality [1–3].

The pathogenesis of NPSLE remains largely unclear, but the occlusion of vessels supplying the nervous tissue and direct interaction of antibodies with phospholipids of neural cells appear to be important. Antiphospholipid antibodies (aPL) may contribute to both of these pathogenic mechanisms [4–6]. aPL are a heterogeneous group of autoantibodies, such as anticardiolipin antibodies (aCL), lupus anticoagulant (LA) and anti-β2-glycoprotein-I (anti-β2GPI), that are frequently observed in autoimmune disorders, especially in SLE. aPL share the ability to bind to phospholipid binding proteins or to complexes of these proteins with phospholipids. β2GPI is the most important aPL antigen [7]. In recent years, the role and relevance of anti-β_2_GPI in autoimmune conditions have been better characterized. The presence of anti-β_2_GPI was included in the list of diagnostic criteria for antiphospholipid syndrome (APS) and, recently, in the Systemic Lupus International Collaborating Clinics classification criteria for SLE [8, 9]. Nonetheless, little is known about the frequency of expression of anti-β_2_GPI in NPSLE, and their role in its pathology.

The aim of the study was to evaluate NPSLE and non-NPSLE patients for the presence of anti-β_2_GPI and other aPL such as aCL and LA and to assess the association between these antibodies and the presence of NPSLE disorders.

## Materials and Methods

### Subjects and diagnostic measures

This study was approved by the local Ethics Committee and conducted in accordance with the Declaration of Helsinki. The study was performed at a university hospital dermatology department, which is a regional reference center for patients with cutaneous and systemic lupus erythematosus. Informed consent was obtained from each participant prior to inclusion into the study. Every patient recruited was interviewed, examined physically by a dermatologist (TH) and blood was sampled. Subsequently, every patient was referred to and examined by a neurologist (AB) and a psychiatrist (MKK).

The examined group comprised 57 consecutive Caucasian SLE in- and out-patients (53 women, 4 men). Each patient met 4 or more ACR classification criteria for SLE [10]. The disease duration was calculated as the time from the first time when at least 4 SLE criteria were fulfilled until the inclusion in the study. Disease activity was assessed once, with the Systemic Lupus Activity Measure (SLAM) score, which ranges from 0 (no disease activity) to 86 (maximum disease activity) [11]. The assessment was performed and material for evaluation of disease activity (blood and urine) sampled during the visit in the department of dermatology. Patients’ corticosteroid doses were calculated for prednisone equivalency. The average corticosteroid dose administered during the last 14 days before examination was defined as the “current daily corticosteroid dose”. Patients’ life cumulative corticosteroid doses were calculated based on their history and medical documentation. A control group of 69 healthy Caucasian volunteers were age and gender matched with the SLE group (62 women, 7 men). The control group had the same blood tests performed as the SLE patients.

### Neurologic and psychiatric assessment

Neurological and psychiatric features of NPSLE were classified according to the current ACR nomenclature, after exclusion of other causes of neuropsychiatric signs and symptoms, defined by ACR as “exclusions” and “associations” [12]. Headaches were classified into one of the three categories: migraine, tension-type headache and cluster headache, which are included in the ACR nomenclature for NPSLE and in the classification of the international headache society (IHS) in the category of primary headaches [12, 13]. Therapy resistant, severe, disabling persistent headache that did not fit any of these categories, after exclusion of secondary causes, was categorized as “intractable headache, nonspecific”. This headache category is included in the ACR classification, but not the IHS classification [12]. Cognitive function was tested in all patients with a Mini Mental State Examination (MMSE) employed as a screening tool [14]. Patients who scored lower than 27 or reported a history of cognitive impairment were further examined neuropsychologically.

### Laboratory measures

All blood samples were collected in a fasting state. Peripheral blood cell counts, erythrocyte sedimentation rate, serum creatinine, and urine were analysed. The presence (dilution > 1:160) and titres of antinuclear antibodies (ANA) were assessed with an indirect immunofluorescence method, by use of a standard diagnostic test, Europlus ANA Mosaic 20 (Euroimmun Medizinische Labordiagnostika AG, Lübeck, Germany) [15]. ANA specificity was determined by the line-blot method with the ANA Euroline Profile 3 kit (Euroimmun Medizinische Labordiagnostika AG, Lübeck, Germany).

The lupus anticoagulant assay was performed according to the recommendations of the Scientific and Standardization Committee of the International Society of Thrombosis and Haemostasis [16]. The assessment was performed on plasma, obtained after centrifugation of the blood sample, collected in a tube with sodium citrate. In the first stage lupus anticoagulant was screened by activated Partial Thromboplastin Time (aPTT) and diluted Russel Viper Venom Time (dRVVT). None of the subjects were being treated with anticoagulants 14 days preceding this testing.

aCL IgG, IgM, IgA and anti-β_2_GPI IgG, IgM, and IgA were assessed with an immunoassay method by a commercial microplate ELISA kit (Euroimmun Medizinische Labordiagnostika AG). Blood samples were obtained by venipuncture (5 ml) from each subject and collected into glass tubes without anticoagulant. The blood was left to clot and then centrifuged. Serum was frozen and stored in aliquots at -80°C degrees. The cut-off value was calculated for each type and class of antibodies at the 98 percentile level of results obtained for the control group, according to previous recommendations [17]. LA, aCL and anti-β_2_GPI assays in SLE patients were performed twice at an interval of at least 12 weeks, according to the current Sydney classification criteria for antiphospholipid antibody syndrome [9]. Only patients who showed aPL (> 98 percentile level of the control group) in both tests were considered positive. In the control group, the antiphospholipid assays were performed only once.

### Statistical analysis

Descriptive statistics including mean, standard deviation, and range were used to present outcomes, and the two tailed Student t-test was used to compare variables with normal distribution. Homogeneity of variance was tested with the Levene’s test. If variances were not homogenous, the Cochran-Cox test was employed. For non-normally distributed variables, medians, upper and lower quartiles and the Mann-Whitney test were used. To compare dichotomic variables, Chi square and whenever appropriate, the Fisher exact test were used. To quantify the strength of the probability of occurrence of neuropsychiatric manifestations in patients with anti-β_2_GPI and other aPL, odds ratio (OR) was used. Statistical significance was set at a level of 0.05.

## Results

### Age of study subjects and SLE duration

The mean age of patients was 42.5 ± 12.2 years (range: 20–69 years), and the mean age of control group subjects was 42.7 ± 10.9 years. The mean SLE duration was 8.3 ± 8.9 years (range: 6 months to 47 years).

### Neuropsychiatric manifestations are frequent and often precede SLE diagnosis

NPSLE was diagnosed in 42 (74%) of 57 consecutive SLE patients. The prevalence of NPSLE manifestations ranged from 39% for headaches to one patient each with acute confusional state and myasthenia gravis (Table 1). In 18 patients only one NPSLE manifestation was diagnosed; most frequently it was headache, which was found, as an isolated manifestation, in 8 patients (14% of SLE patients), 6 (11%) of whom had migraine headaches. Two further patients were not included in the NPSLE-headache group as their headaches were associated with seizures and diagnosed as ictal headaches.

**Table 1:** Prevalence of neuropsychiatric systemic lupus erythematosus (NPSLE) in patients with systemic lupus erythematosus (SLE).

	Number of subjects (percentage of all SLE patients, N = 57)
non-NPSLE	15 (26.3%)
NPSLE	42 (73.7%)
Headache	22 (38.6%)
Migraine	10 (17.5%)
Tension headache	2 (3.5%)
Nonspecific, intractable headache	10 (17.5%)
Cerebrovascular disease (CVD)	13 (22.8%)
Ischaemic stroke	10 (17.5%)
Transient ischemic attack (TIA)	3 (5.3%)
Intracranial hemorrhage	1 (1.8%)
Seizures	8 (14.0%)
Grand mal seizures	6 (10.5%)
Partial simple seizures	2 (3.5%)
Partial complex seizures	1 (1.8%)
Multiple mononeuropathy	6 (10.5%)
Single mononeuropathy	1 (1.8%)
Sensory polyneuropathy	3 (5.3%)
Cranial neuropathy (of the 5th and the 8th nerve)	2 (3.5%)
Myasthenia gravis	1 (1.8%)
Acute confusional state	1 (1.8%)
Cognitive dysfunction	5 (8.8%)
Depressive disorders	11 (19.3%)
Anxiety disorders	6 (10.5%)

**Fig 1:**
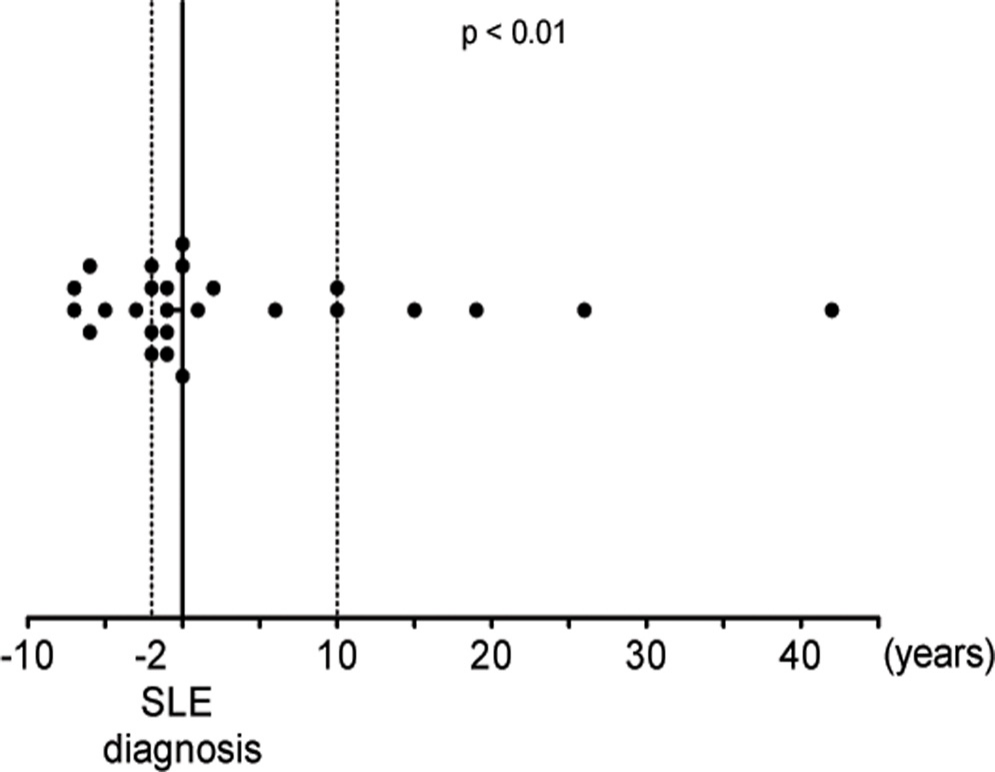
Relation of diagnosis of SLE and the onset of neuropsychiatric symptoms. Values are negative in those patients, who developed neuropsychiatric symptoms before they were diagnosed with SLE. Dotted lines show the median intervals between the onset of neuropsychiatric symptoms and diagnosis of SLE, i.e. 2 years in patients who developed neuropsychiatric symptoms before they were diagnosed with SLE and 10 years in patients who developed neuropsychiatric symptoms after they were diagnosed with SLE.

In 26 patients it was possible to assess the temporal relationship of the first neuropsychiatric symptoms and SLE diagnosis. In 17 (65.4%) of these NPSLE patients, neuropsychiatric symptoms preceded SLE diagnosis, and in 9 (34.6% of NPSLE patients) the onset of symptoms followed the diagnosis of SLE. In the 17 patients who showed neuropsychiatric symptoms before they were diagnosed with SLE, the median interval was 2 years (lower quartile: 1; upper quartile: 5.5). This was significantly (p<0.01) shorter than the median interval of 10 years (lower quartile: 4; upper quartile: 22.5) in patients who developed neuropsychiatric manifestations after they had been diagnosed with SLE (Fig. 1).

### NPSLE and non-NPSLE patients show comparable SLE activity, corticosteroid use, antiphospholipid antibody profiles, and frequency of APS

**Fig 2:**
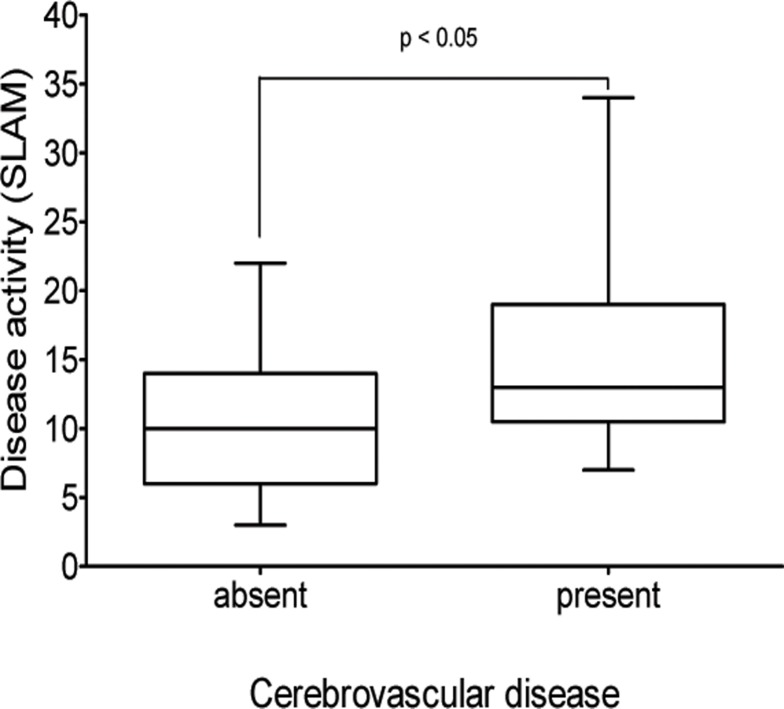
Comparison of systemic lupus erythematosus (SLE) activity, as measured with the Systemic Lupus Activity Measure (SLAM), in patients with and without cerebrovascular diseases. Median values expressed as horizontal lines, interquartile ranges as boxes, and ranges as whiskers.

The mean disease activity in our SLE patients was 11.3 ± 6.0, range 3–34, as assessed by SLAM. We did not find a significant difference in the mean SLE activity between NPSLE and non-NPSLE patients. The only NPSLE subgroup that exhibited increased disease activity was patients with cerebrovascular diseases (CVD): median disease activity in these patients was significantly higher (13.0; lower quartile 10.5, upper quartile 19.0) than in patients without cerebrovascular diseases (10.0; lower quartile 6.0, upper quartile 14.0; p<0.05) (Fig. 2). We did not observe any statistically significant differences in the frequency of ACR criteria for SLE between NPSLE and non-NPSLE patients (Table 2). The mean current daily corticosteroid dose of all patients was 11.2 ± 13.8 mg (range 0–60 mg), and the mean life cumulative corticosteroid dose was 25.4 ± 32.3 g (range 0–123 g). There was no difference between NPSLE and non-NPSLE groups in corticosteroid doses, neither current nor cumulative.

**Table 2:** Clinical and laboratory characteristics of NPSLE and non-NPSLE patients according to the American College of Rheumatology (ACR) classification criteria.

ACR criteria	NPSLE N = 42 (%)	non-NPSLE, N = 15 (%)
Malar rash	34 (81.0)	10 (66.7)
Discoid rash	10 (23.8)	4 (26.7)
Photosensitivity	38 (90.5)	11 (73.3)
Oral ulcers	22 (52.4)	4 (26.7)
Arthritis	38 (90.5)	14 (93.3)
Serositis	3 (7.1)	3 (20.0)
Renal disorder	13 (31.0)	7 (46.7)
Hematologic disorder	34 (81.0)	13 (86.7)
hemolytic anemia	19 (45.2)	8 (53.3)
leukopenia	27 (64.3)	10 (66.7)
lymphopenia	14 (33.3)	8 (53.3)
thrombocytopenia	17 (40.5)	4 (26.7)
Immunologic disorder	23 (54.8)	8 (53.3)
anti-dsDNA antibodies	8 (19.0)	3 (20.0)
anti-Sm antibodies	8 (19.0)	2 (13.3)
VDRL—false positive	1 (2.3)	1 (7.1)
anticardiolipin antibodies IgG or IgM	11 (26.2)	3 (20.0)
lupus anticoagulant	5 (11.9)	2 (13.3)
ANA positive (titer > 1/160)	42 (100)	15 (100)

NPSLE—neuropsychiatric systemic lupus erythematosus; ANA—antinuclear antibodies

Differences between NPSLE and non-NPSLE groups are statistically insignificant.

Antiphospholipid antibodies, i.e. IgG, IgM, or IgA antibodies against β_2_GPI or CL as well as LA, were found in 11% to 26% of patients (Table 3). There were no significant differences of the rates of any of these aPL between NPSLE patients and non-NPSLE patients.

**Table 3:** Prevalence of antiphospholipid antibodies in patients with systemic lupus erythematosus (SLE) and in the subgroups with and without neuropsychiatric (NP) manifestations.

Antiphospholipid antibodies		SLE, N = 57	NPSLE, N = 42	non-NPSLE, N = 15
Anti-β2glycoprotein antibodies	IgG	8 (14.0%)	5 (11.9%)	3 (20%)
	IgM	9 (15.8%)	8 (19.0%)	1 (6.7%)
	IgA	6 (10.5%)	4 (9.5%)	2 (13.3%)
Anticardiolipin antibodies	IgG	6 (10.5%)	4 (9.5%)	2 (13.3%)
	IgM	9 (15.8%)	8 (19.0%)	1 (6.7%)
	IgA	15 (26.3%)	11 (26.2%)	4 (26.7%)
Lupus anticoagulant		7 (12.3%)	5 (11.9%)	2 (13.3%)

Differences between NPSLE and non-NPSLE groups are statistically insignificant.

13 of all SLE patients (23%) were previously diagnosed with APS. 12 of these 13 patients were confirmed to be positive for aCL in the current examination. Diagnosis of APS was not related to any of the NPSLE disorders, with the exception of CVD, and ischemic stroke specifically, which itself is a clinical criterion of APS.

### Patients with anti-β_2_GPI present more frequently with headaches, ischemic stroke or seizures, and seizures are also linked to aCL and LA

When we compared patients with certain aPL for differences in their NPSLE manifestations we found that patients with anti-β_2_GPI IgM have an almost six times higher likelihood of having nonspecific, intractable headaches (Table 4) or ischemic stroke (Table 5) than patients negative for these antibodies. Patients with anti-β_2_GPI IgG were found to be 11 times more likely to exhibit seizures (Table 6) and 9 times more likely to have grand mal seizures specifically (Table 7) than patients negative for these antibodies. We found seizures to also be linked to LA and aCL IgA (Table 6), and grand mal seizures to aCL IgA only (Table 7). The remaining NPSLE subgroups showed no apparent association with the investigated antibodies.

**Table 4:** Association of antiphospholipid antibodies with nonspecific headaches.

	Headaches nonspecific	Fisher exact test, p value	OR	95% CI
anti-β_2_GPI IgM (+) n = 9	4 (44.4%)	< 0.05	5.60	1.17–26.88
anti-β_2_GPI IgM (-) n = 48	6 (12.5%)			

anti-β_2_GPI—anti-β2-glycoprotein-I; IgM—immunoglobulin M; OR—odds ratio; CI—confidence interval

**Table 5:** Association of antiphospholipid antibodies with ischemic stroke.

	Ischemic stroke	Fisher exact test, p value	OR	95% CI
anti-β_2_GPI IgM (+) n = 9	4 (44.4%)	< 0.05	5.60	1.17–26.88
anti-β_2_GPI IgM (-) n = 48	6 (12.5%)			

anti-β_2_GPI—anti-β2-glycoprotein-I; IgM—immunoglobulin M; OR—odds ratio; CI—confidence interval

**Table 6:** Association of antiphospholipid antibodies with seizures.

	Seizures	Fisher exact test, p value	OR	95% CI
anti-β_2_GPI IgG (+) n = 8	4 (50.0%)	0.010	11.25	2.01–62.97
anti-β_2_GPI IgG (-) n = 49	4 (8.2%)			
LA (+) n = 7	3 (42.9%)	0.05	6.75	1.16–39.20
LA (-) n = 50	5 (10.0%)			
aCL IgA (+) n = 15	5 (33.3%)	< 0.05	6.50	1.32–31.91
aCL IgA (-) n = 42	3 (7.1%)			

anti-β_2_GPI—anti-β2-glycoprotein-I; aCL—anticardiolipin; IgG—immunoglobulin G; IgA—immunoglobulin A; LA—lupus anticoagulant; OR—odds ratio; CI—confidence interval

**Table 7:** Association of antiphospholipid antibodies with grand mal seizures.

	Grand mal seizures	Fisher exact test, p value	OR	95% CI
anti-β_2_GPI IgG (+) n = 8	3 (37.5%)	< 0.05	9.20	1.45–58.36
anti-β_2_GPI IgG (-) n = 49	3 (6.1%)			
aCL IgA (+) n = 15	4 (26.7%)	< 0.05	7.27	1.17–45.06
aCL IgA (-) n = 42	2 (4.8%)			

anti-β_2_GPI—anti-β2-glycoprotein-I; aCL—anticardiolipin; IgG—immunoglobulin G; IgA—immunoglobulin A; OR—odds ratio; CI—confidence interval

## Discussion

Here, we characterized the prevalence, disease activity, onset and aPL profiles of patients with NPSLE. Most importantly, we demonstrate, to our knowledge for the first time, that specific neuropsychiatric manifestations in SLE, namely intractable headaches, ischemic stroke and seizures are linked to the presence of anti-β2GPI. Notably, anti-β2GPI were the only or the most predictive antibodies for all of these manifestations.

The overall prevalence of NPSLE in our patients was high and consistent with data from studies using the same ACR nomenclature [18–23]. Ainiala as well as Hanly and co-workers proposed stricter criteria excluding manifestations which relationship with SLE pathology is controversial [24–26]. Here, we decided to include all ACR neuropsychiatric manifestations, to further test their relationship with objective clinical and laboratory findings including aPL.

We observed, consistent with previous findings, that the onset of neuropsychiatric manifestations preceded SLE diagnosis in the majority of cases, and at the same time it took many years for the other patients to develop NPSLE [27, 28]. This indicates the need to educate clinicians to consider SLE in patients who show neurologic manifestations, but also to continuously monitor SLE patients for symptoms of NPSLE. It may be speculated that immunosuppressive therapy, which is often started once the diagnosis of SLE is established, might delay progression to NPSLE, resulting in its delayed onset.

We did not find any differences in disease activity and corticosteroid use comparing NPSLE and non-NPSLE patients. Analyzing individual NPSLE manifestations separately, only CVD was linked to a higher SLE activity. We also did not find any differences in the frequency of ACR criteria for SLE between NPSLE and non-NPSLE patients, including hematologic criteria, which were previously reported to be related to NPSLE [27, 29].

Positivity to aCL appeared to be stable in patients with APS, and almost all patients with pre-existing APS appeared to be positive to aCL. Frequency of APS did not differ between NPSLE and non-NPSLE patients.

In our patients, NPSLE was not related to differences in aPL expression, and this is in line with previous studies that used the same diagnostic criteria we did [20, 30].

Anti-β2GPI were demonstrated in the 1990s to play a crucial role in APS [7, 31]. In contrast, the role of anti-β2GPI in SLE including NPSLE is less well characterized and understood [32]. As of now, there are only a few studies that have investigated anti-β2GPI in NPSLE, and all of them failed to show that anti-β2GPI are associated with any of the individual NPSLE manifestations [19, 25, 33–37]. Importantly, we found specific NP manifestations of SLE, namely intractable headaches, ischemic stroke and seizures to be linked to the presence of anti-β2GPI and other aPL.

The attribution of headaches to NPSLE was previously questioned and remains controversial [26, 38–40]. In accordance with most but not all previous reports [36, 40–42] we did not find a link between the occurrence of headaches and SLE activity. Interestingly, we found that headaches classified according to the ACR nomenclature as intractable, nonspecific were present in 18% of our patients, and they were associated with anti-β_2_GPI IgM. This suggests that intractable headaches can be a manifestation of NPSLE and that anti-β_2_GPI IgM may be useful in their diagnostic workup. In contrast to a previous report [43], we did not observe an association of aPL with migraine, which confirms other recent studies, including prospective ones [44, 45]. The most likely reason for the fact that the majority of previous studies did not find headaches to be linked to aPL is that these studies only looked at all headaches as a group or at migraine but not other subtypes of headache [36, 40, 41, 46, 47]. Interestingly, five APS patients with intractable headaches were previously reported to benefit from anticoagulation therapy and to relapse after its cessation [48].

In SLE, a link between CVD and aPL was previously confirmed for LA [19, 25, 49, 50] and IgG [22, 49–51] as well as IgM class aCL [22]. We found ischemic stroke to be associated with anti-β_2_GPI, which is in line with a previous study in children with SLE and APS [52]. Stroke is the second most frequent vascular thrombosis, after deep vein thrombosis, and it is the most frequent presentation of arterial thrombosis in APS [53, 54]. Seizures are a well known manifestation of NPSLE, included also in the list of ACR criteria for SLE [10]. Epilepsy is also associated with APS, and as many as 10% of patients with primary APS develop seizures [55]. Patients with epilepsy are more frequently positive for aCL and anti-β2GPI [56–58].

In SLE, an association between seizures and IgG [22, 50, 59, 60] and IgM class aCL [22] was reported in some studies but not others [19, 33, 49, 51]. For LA, Herranz and co-workers found 44% of SLE patients with epilepsy to be positive, as compared to 43% in our study [60]. We failed to find any study that previously investigated IgA aCL in NPSLE. Interestingly, there is one study that tested and confirmed an association between seizures and IgA aCL in APS, and its results are consistent with our results in NPSLE, indicating a possible role of IgA aCL in the pathogenesis of seizures and confirming the importance of IgA isotype testing [58].

### Strengths and limitations

Our study has several strengths and limitations. Most previous studies on aPL in NPSLE relied on single aPL measurements whereas we performed two independent measurements at least 12 weeks apart, in accordance with current diagnostic laboratory criteria for APS [9]. Single antibody measurements may result in decreased specificity, leading to classify as positive also transiently positive results [61]. ELISA assays for aPL and anti-β2GPI in particular are still inadequately standardized [62, 63], and the use of local cut-off values is recommended [17, 64]. These cut-off values should be based on control values from age matched subjects, as children and elderly may express higher serum levels of aPL [65, 66]. We employed this approach to minimize this known problem.

The limitations of our study include the limited number of SLE patients. Further studies on larger samples of SLE patients are required to draw firm conclusions on the associations of autoantibodies with specific NPSLE manifestations, especially to investigate rare NPSLE disorders. Secondly, we did not reassess the expression of aPL and neuropsychiatric signs and symptoms after many years, which is needed to learn about the long term influence of persistent aPL. Finally, cognitive impairment was, differently than recommended by ACR criteria for NPSLE, screened with MMSE and clinical history only, instead of a battery of neuropsychological tests, which may have resulted in lower sensitivity.

## Conclusions

In summary, our results confirm a high prevalence of NPSLE in SLE patients and show that neuropsychiatric manifestations often precede SLE diagnosis. Most importantly, we found a link between anti-β2GPI and non-specific, intractable headaches, ischemic stroke and seizures, and these autoantibodies show a better predictive value than other aPL such as aCL and LA. Further studies on the role and relevance of persistent anti-β2GPI in headaches, ischemic stroke, and seizures are needed and may elucidate pathogenic effects of anti-β2GPI as possible, novel targets for more effective therapies of NPSLE.
